# Model for Achieving a Coordinated Access to Lung Cancer Care in Selected Public Health Facilities in KwaZulu-Natal, South Africa: Protocol for a Qualitative Study

**DOI:** 10.2196/34341

**Published:** 2023-03-03

**Authors:** Buhle Lubuzo, Khumbulani Hlongwana, Themba Ginindza

**Affiliations:** 1 Discipline of Public Health Medicine School of Nursing and Public Health University of KwaZulu-Natal Durban South Africa

**Keywords:** Africa, cancer care, cancer, coordination model, KwaZulu-Natal, lung cancer, lung, low income, middle income, LMIC, care access, care coordination, coordinated care, care model, cost, health care system, oncology

## Abstract

**Background:**

Timely delivery of high-quality cancer care to all patients is barely achieved in South Africa and many other low- and middle-income countries, mainly due to poor care coordination and access to care services. After health care visits, many patients leave facilities confused about their diagnosis, prognosis, options for treatment, and the next steps in their care continuum. They often find the health care system disempowering and inaccessible, thereby making access to health care services inequitable, with the resultant outcome of increased cancer mortality rates.

**Objective:**

The aim of this study is to propose a model for cancer care coordination interventions that can be used to guide and achieve coordinated access to lung cancer care in the selected public health care facilities in KwaZulu-Natal.

**Methods:**

This study will be conducted through a grounded theory design and an activity-based costing approach that will include health care providers, patients, and their caregivers. The study participants will be purposively selected, and a nonprobability sample will be selected based on characteristics, experiences of the health care providers, and the objectives of the study. With the study’s objectives in mind, communities in Durban and Pietermaritzburg were selected as study sites, for the study along with the 3 public health facilities that provide cancer diagnosis, treatment, and care in the province. The study involves a range of data collection techniques, namely, in-depth interviews, evidence synthesis reviews, and focus group discussions. A thematic and cost-benefit analysis will be used.

**Results:**

This study receives support from the Multinational Lung Cancer Control Program. The study obtained ethics approval and gatekeeper permission from the University’s Ethics Committee and the KwaZulu-Natal Provincial Department of Health, as it is being conducted in health facilities in KwaZulu-Natal province. As of January 2023, we had enrolled 50 participants, both health care providers and patients. Dissemination activities will involve community and stakeholder dissemination meetings, publications in peer-reviewed journals, and presentations at regional and international conferences.

**Conclusions:**

This study will provide comprehensive data to inform and empower patients, professionals, policy architects, and related decision makers to manage and improve cancer care coordination. This unique intervention or model will address the multifactorial problem of cancer health disparities. If successful, this study will affect the design and implementation of coordination programs to promote optimal cancer care for underserved patients.

**International Registered Report Identifier (IRRID):**

DERR1-10.2196/34341

## Introduction

### Background Information

Cancer is increasingly becoming one of the leading public health problems globally [1-4]. The World Health Organization’s projections indicate that global annual estimates will increase to 29.5 million new cancer cases and 16.5 million cancer-related deaths by the year 2040 [2,3,5]. Of concern is the fact that the current evidence demonstrates that survival from all cancers, including preventable lung cancer, is poor [2-10], and this is partly reflective of the poor access to coordinated cancer care. The public health challenges of the escalating cancer burden are more pronounced in low- and middle-income countries (LMICs) [9]. Patients with cancer in these countries are often diagnosed late (at stage 3 and 4) and the pathways of care are not clearly defined, thus delaying treatment initiation and leading to disproportionately high mortality rates and reduced quality of life when compared to their higher-income counterparts [5,9,11-13].

Medical care is error-prone even when it is delivered by a single provider; the opportunities for serious issues or difficulties escalate when multiple providers are involved [14-21]. Inherent care complexities for patients with lung cancer make these patients likely candidates for shared care between primary care providers, oncologists, and other specialties. A study investigating barriers impeding quality care coordination for patients with lung cancer in South Africa, from the perspectives of the health care providers, identified these challenges as contributing to adverse outcomes in lung cancer care [14]. Issues relating to access, timely delivery, and coordination of care ranked the highest in the priority list shared by health care providers [22]. These issues appeared to facilitate the rising numbers of cancer fatalities in South Africa [14]. Evidence from South Africa is consistent with what has been found in other countries [13,23-25], asserting that when a diagnosis is accurate and timely, a patient has an increased opportunity for a positive health outcome [26-37].

Despite the potential benefits of patient access to coordinated cancer care services [26-37], most countries in LMICs, such as South Africa, do not have systems for achieving this coordination. Specifically, lung cancer diagnosis has far-reaching and life-changing consequences for patients [14,16,20,36,38-42], with the disease likely to be at an advanced stage at the time of detection [14,16,43]. In South Africa, the structural configuration of the health systems compounds the challenges relating to providing quality and coordinated cancer care [44]. These challenges include socioeconomic factors, the unavailability of technical support for diagnosis and disease staging, the initiation of and lack of resources for treatment, and the poor availability of palliative care and other support services [14,16,20,21,44-48]. The current referral pathways for patients with cancer between the health care team and cancer services are either not structured or are poorly documented [13,14,47]. Patients with cancer often have limited familiarity with the multidisciplinary team involved and therefore lack the specific knowledge and resources needed to navigate the specialists and services involved in their clinical care. In turn, each team brings varying perspectives to the care of the same patient, whose true value can be achieved through proper communication. The interface between primary and specialty care for patients struggling with cancer offers a valuable opportunity to appreciate the challenges of delivering well-coordinated care.

To be comprehensive, cancer care must be coordinated between primary care providers and oncology specialists; however, achieving this has proven to be considerably challenging [14-21]. Understanding the strategies that can be used to achieve this coordination is necessary for better cancer care. However, attempts to improve care coordination can only be achieved through a properly and intentionally designed care coordination intervention strategy [49]. The high inconsistency of practices, the diversity of definitions, and the underlying concepts [49-52] increase the current difficulty to standardize, replicate, transpose, and assess care coordination, especially within the South African health system context. As such, the aim of this study is to propose a model for cancer care coordination interventions that can be used to guide and achieve coordinated access to lung cancer care in the selected public health care facilities in KwaZulu-Natal, South Africa.

### Study Aim and Objectives

This study aims to propose a model for cancer care coordination interventions that can be used to guide and achieve coordinated access to lung cancer care in the selected public health care facilities in KwaZulu-Natal. This will be carried out in 4 phases. The specific objectives are as follows:

*Phase 1: Assess the factors that affect access or delivery of coordinated lung cancer care*

- To identify health system barriers and key pillars affecting access to a coordinated lung cancer care continuum.
- To conduct a needs assessment to lung cancer care necessary for achieving coordinated care.

*Phase 2: Identify a model relative to cancer care coordination interventions*

- To understand the scope of cancer care coordination interventions and services employed in low-and middle-income countries, in order to synthesize the existing evidence and identify the key model and its elements used to manage and improve cancer care coordination in these settings.
- To systematically analyze the available evidence on the identified model’s characteristics, outcomes, and effectiveness across the cancer care continuum in low-and middle-income countries.

*Phase 3: Identify key elements of the proposed model*

- To explore what the primary functions of proposed model will be.
- To identify the roles and skill sets needed for proposing the key personnel required to implement the proposed model.
- To explore how the proposed model can be better integrated into the clinical care program.

*Phase 4: Costing of the proposed intervention model*

- To conduct a costing exercise in order to determine the feasibility of developing and implementing the proposed model.

## Methods

### Study Design

The study will use a mixture of the grounded theory (GT) approach and an activity-based costing (ABC) approach. The central principle of GT is that the researcher’s theories about a topic are constructed based on their data [53]. It is appropriate when little is known about a phenomenon; the aim being to produce or construct an explanatory theory that uncovers a process inherent to the substantive area of inquiry [53-55]. GT sets out to discover or construct theory from data that has been systematically obtained and analyzed using comparative analysis. The study will predominantly be conducted through qualitative research methods, using participatory approaches. This is founded on the principles of active citizenry and constructivist theory, which views research participants as co-constructors of knowledge and not just passive subjects [56]. As a result, the study falls within the constructivism paradigm (interpretivist), as it aims to explore and understand the participants’ experiences [57].

Described as “the most comprehensive qualitative research methodology available” [58], this approach is appropriate when research aims to explain a process where the concerns of those involved are central to its understanding and cannot be predetermined [55,58-60]. GT uses memoing or memos, which are ideas generated and documented through interacting with data to provide detailed records of the researchers’ thoughts, feelings, and intuitive contemplations [54]. The theoretical sensitivity encompasses the entire research process; it is the ability to know when you identify a data segment that is important to your theory [54].

The ABC approach has 2 major elements: cost measures and performance measures. It is a methodology that measures the cost and performance of activities, resources, and cost objects [61]. Resources are assigned to activities, and then activities are assigned to cost objects based on their use. The basic concept of ABC is that activities consume resources to produce an output. It assists to identify activities in a project or organization and assigns the cost of each activity to all products and services according to the actual consumption by each [61]. This approach uses both financial and nonfinancial variables as bases for cost allocation. Today’s health care system encompasses a wide variety of services. To manage them, it is important to determine the amount of resources that are consumed by each service. The complexity of widely varied service delivery systems can be readily managed with ABC, as its methodology is particularly suited to the complexities of health care service delivery [61].

### Study Area

The study will be conducted in the province of KwaZulu-Natal. This province has the second-largest economy in the country [62]. The study will involve 2 cities, namely, Durban and Pietermaritzburg, which are within the KwaZulu-Natal province. KwaZulu-Natal has 1 metropolis and 10 districts. While Pietermaritzburg is the capital city of the province, Durban is the largest city in the province [63]. Durban and Pietermaritzburg have an estimated population of 3,120,282 and 750,845, respectively [64]. With the objectives of the study in mind, communities in Durban and Pietermaritzburg were selected as study sites, along with 3 health facilities that provide cancer diagnosis, treatment, and care in the province. In total, 3 of the communities are in Durban, and 2 are in Pietermaritzburg. These communities are Umlazi, Chatsworth, and South Durban Basin in Durban, and Imbali and Sobantu in Pietermaritzburg.

### Study Settings

A geographical scoping and mapping of study clusters were done in each of the representative communities. In total, 40 out of 879 clusters were selected using probability proportional to population size sampling. Maps were used to identify the selected clusters along with their boundaries. This technical mapping exercise gave details of the location of the communities, population numbers, broad socioeconomic baseline characteristics of the communities, and resources available in the area, for example, clinics and hospitals, as well as other community social commons.

We selected both clinics and hospitals to ensure that the sampled data have implications for the full spectrum of cancer care providers. Furthermore, all public hospitals with oncology units in KwaZulu-Natal (Greys Hospital, Addington Hospital, and Inkosi Albert Luthuli Central Hospital) located in 2 cities will be recruited to participate in the study. These 3 participating health facilities were chosen on the basis that they are the only public hospitals offering oncology services in the province. Greys Hospital is located in Pietermaritzburg, whereas Inkosi Albert Luthuli Central Hospital and Addington Hospital are located in Durban.

### Data Collection Process

Research staff will be concerned about participants’ risks and also their risk of exposure during the collection of data. The study will be conducted in 4 phases.

Phase 1 will be through in-depth interviews (IDIs), determining the barriers and pillars affecting coordinated lung cancer care. Accomplishments and ongoing barriers to engagement in medical care identiﬁed in phase 1 will be prioritized and translated into service plans that will deﬁne the speciﬁc action steps in the proposed model. Throughout this phase, participants’ demographic and clinical information will be collected.

Phase 2 will focus on identifying an intervention aimed at assisting patients in overcoming barriers to coordinated lung cancer care in KwaZulu-Natal. Based on phase 1 results, the models will be reviewed and modified to meet the needs identified in phase 1. The following will be key areas of focus for the strategy during the scale-up process. This phase will be conducted in two steps: (1) a scoping review to identify relevant models from the literature and all their components for coordinated care and (2) a systematic review of the identified or proposed care coordination model’s effectiveness. The aim of this phase is to provide descriptions of a wide variety of care coordination interventions and thereafter propose an intervention or model. The search strategy will include PubMed, Scopus, Google Scholar, and Web science databases using keywords such as “cancer care coordination,” “coordination of care,” or “coordinated care.”

Phase 3 will be through focus group discussion (FGD). The approach will facilitate awareness, understanding, and commitment to the optimal proposed cancer care coordination model among stakeholders and facilities involved. The goal is to identify key elements of the proposed model and obtain stakeholder support for coordinated referrals and linkage pathways for patients with cancer. This is anticipated to inform the development and implementation of a coordination model to improve lung cancer care.

Lastly, in phase 4, a costing exercise will be conducted in order to determine the feasibility of the development and implementation of the proposed model; this may be useful in the move to certify and establish the intervention. This phase will be achieved through ABC.

### Data Collection Tools

For this study, an IDI and FGD guide will be used to address the study objectives. These were developed for this study; however, the FGD guide will be informed by the IDIs’ and phase 2 responses. Both the IDIs and FGDs will be facilitated by the lead investigator, who is experienced in qualitative research. Lastly, the costing exercise will be informed by findings from the IDIs, FGDs, and reviews. Table 1 provides study data collection methodology by phases.

**Table 1:** Summary of data collection methodology.

Tools and data source				Variables		Analysis plan
**Phase 1: assess factors that affect coordinated access to lung cancer care (interviews)**						
	Patients and caregivers			Description of their care, barriers, and how it was managed When the diagnosis was made How the diagnosis was confirmed Treatment initiation Reasons for not initiating treatment Time of treatment initiation Understanding of “care coordination” Suggestions to improve care coordination		GT^a^ approach analysis
	Health care providers			Description of what “care coordination” services involve Outline of their roles in a patient’s “care coordination” Essentials of “care coordination” Weaknesses in “care coordination” processes Suggestions to improve care coordination		GT approach analysis
**Phase 2: identify model relative to cancer care coordination interventions**						
	**Scoping review**					
		Scopus, PubMed, and Google Scholar	Relevant models and all its components relevant for coordinated care		PRISMA^b^ guide	
	**Systematic review**					
		Scopus, PubMed, and Google Scholar	Informed by the scoping review		PRISMA guide	
**Phase 3: identify key elements of proposed model (focus group discussion)**						
	Identified stakeholders and health care professionals to be involved in the designing of the proposed model			What will be the primary functions of the proposed model? What will they fulfill? Outline of their roles in a patient’s “care coordination” What additional roles and skills set needed How model can be integrated into the current clinical cancer care		Thematic analysis
**Phase 4: costing of proposed intervention model (ABC^c^ approach)**						
	Variables included in phases 1, 2, and 3			Human resource costs for recruitment and hiring Operational costs Capital equipment costs Model specific training costs		Costing analysis

^a^GT: grounded theory.

^b^PRISMA: Preferred Reporting Items for Systematic Reviews and Meta-Analyses.

^c^ABC: activity-based costing.

### Study Population

The study population will encompass patients, health care providers drawn from the participating health care facilities, including surgeons, radio-oncologists, medical oncologists, general practitioners, chemotherapy nurse practitioners, and social workers, all with a coordination activity for patients with cancer, and also the caregivers with whom they interact as part of their care activity. Caregivers will be proxies for patients who are unable or not in a condition to answer the questions and participate in the study. A proxy is a person authorized to act on behalf of someone else [65].

#### Inclusion and Exclusion Criteria

There are no exclusion criteria based on socioeconomic status, race, or sex, but patients with no lung cancer will be excluded. Textbox 1 summarizes inclusion and exclusion criteria for study participants by phases.

**Inclusion and exclusion criteria for study participants by phases.**

**Phase 1: patients with lung cancer (all stages), caregivers, and health professionals in lung cancer care**

**Inclusion criteria**

- Patients diagnosed with lung cancer and are within the selected facilities
- Caregivers of patients who meet the inclusion criteria and are unable but willing to participate in the study
- Health professionals involved in the care and coordination of patients with cancer

**Exclusion criteria**

- Patients diagnosed with other diseases other than lung cancer
- Patients receiving care for lung cancer, but outside the selected facilities
- Caregivers of patients with lung cancer who do not meet the inclusion criteria
- Professionals who are not involved in the care of patients with cancer

**Phase 2: systematic and scoping review**

**Inclusion criteria**

- Studies presenting evidence published in low- and middle-income countries (LMICs)
- Studies presenting evidence on cancer care coordination
- Studies presenting evidence on coordination interventions or models
- Studies reported in English

**Exclusion criteria**

- Studies presenting evidence not published in LMICs
- Studies not presenting evidence on cancer care coordination
- Studies presenting evidence on coordination interventions or models
- Studies reported in English

**Phase 3: identified stakeholders to be involved in the designing of the proposed model for coordination**

**Inclusion criteria**

- Identified stakeholders to be involved in the designing of the proposed model for coordination
- Health professionals involved in the care activity of patients with cancer

**Exclusion criteria**

- Stakeholders with no coordination activity for patients with lung cancer
- Health professionals with no role in the care activity of patients with cancer

**Phase 4: informed by phases 1, 2, and 3**

### Recruitment Procedure

Eligible patients will be identified during their visits or hospitalization in the participating facilities and will be informed about the study in a personal interview and with the help of written consent documents. A modified version of snowballing will be used to identify caregivers of patients not eligible to participate due to health conditions. Patients will be asked to invite their primary caregivers to participate in the study. Health care providers involved in cancer care (including surgeons, radio-oncologists, medical oncologists, general practitioners, chemotherapy nurse practitioners, and cancer nurse coordinators) will be approached directly by the research team to participate in a focus group or interview. Eligible potential participants will be invited by email or telephone to participate in the study. The invitation letter will include a general introduction to the research topic and the study’s aim and rationale. They will also be presented with information about their involvement, such as the anticipated length of the interview and where it would be conducted. General information about measures taken to guarantee confidentiality and informed consent will also be given.

### Sampling Strategy

#### In-depth Interviews

Purposive sampling will be used to identify the participants relevant to address the research objectives [53-55,60]. Participants will be selected based on their characteristics, experiences, and the objectives of the study [66]. Concurrent data generation and analysis are fundamental to GT research design [67]. Hence, data generation, data coding, and analyses will be done iteratively. Theoretical sampling will begin with the codes and categories developed from the first data set. Theoretical sampling is used to identify and follow clues from the analysis, fill gaps, clarify uncertainties, check hunches, and test interpretations as the study progresses [67]. The selection of the study sample will be done per study site and according to the study phases and objectives.

#### Focus Group Discussions

The sample for FGDs will be purposive and will include individuals with characteristics of the overall population. Purposive sampling is often used when a researcher wants information-rich participants [6], in the case of FGD, a sub-sample of insightful participants from the phase 1 interviews will be invited for discussion. Each focus group will have a professional or stakeholder representing all levels of the health care systems.

### Sample Size

For the IDIs, sampling saturation will be achieved when the same stories, themes, or codes and issues are emerging from the interviewees, and then a sufficient sample size will have been reached (saturation). We will take note of the importance of availability (limited number of health providers working within the oncological units), willingness, and the ability to communicate experiences and opinions in an articulate, expressive, and reflective manner. It is anticipated that data saturation will be reached at 25 interviews for each category (patients and professionals) of participants. For FGDs, and with support from the literature, the sample size will be between 7 and 12 participants to be effective [68].

### Data Quality Assurance

#### Internal Validity

Bias can arise from the approach or instrument adopted for collecting or measuring data in a study [69]. Information bias, otherwise known as misclassification, is one of the most common sources of bias affecting health research’s validity [63]. This bias will be minimized through the extensive training of the research assistants. The questionnaires will be validated and standardized during the training. To ensure the quality of the translation, an experienced translator competent in both English and isiZulu will translate the questionnaires, and a different person will perform the back-translation. The principal investigator will compare the original copy with the back-translation, and where necessary, adjustments will be made.

#### Measuring the Trustworthiness of the Qualitative Data

Credibility is defined as the confidence that can be placed in the truth value of the research findings, which is essential in establishing whether or not the research findings represent plausible information drawn from the participants’ original data and is a correct interpretation of the participants’ original views [70]. In this study, credibility will be ensured through the search for alternative themes developing from the data and the use of verbatim quotes when reporting the findings. In certain circumstances, where the translation will diminish the original meaning, isiZulu quotes may be retained with explanatory text. The researchers who conduct the IDIs with the key informants will receive advanced training on interviews and other qualitative research processes in order to ensure the collection of high-quality data. Analyst triangulation will be performed, whereby the principal investigator and the supervisors will analyze the transcripts independently and later discuss the analysis. According to Bitsch [71], dependability refers to “the stability of findings over time.” Dependability will be ensured through a clear exposition of data collection and analysis methods and also through data source triangulation (with various key informants).

The study results will be based on the data generated through the thick description (ie, a rich and extensive set of details) of the phenomenon in order to enhance the transferability of the findings. The thick description will also include the context in which the study was carried out. Transferability, a concept that is comparable to generalizability in quantitative research, refers to the degree to which the results of qualitative research can be transferred to other settings with comparable contexts [71]. Confirmability will be ensured by keeping an audit trail, carrying out source and data triangulation, and also carrying out peer review or debriefing. Confirmability refers to the degree to which the results of an inquiry could be confirmed or corroborated by other researchers [72].

### Data Analysis

Data analysis will follow a GT approach, a constant comparative analysis [54,73]. In GT-based analysis, the researcher generally analyzes the data by (1) finding repeating themes by thoroughly reviewing the data, (2) coding the emergent themes with keywords and phrases, (3) grouping the codes into concepts hierarchically, and then (4) categorizing the concepts through relationship identification [73-76]. Finally, the categories created through this process, and the links found between them, are used as the basis for the development of a new theory [74]. These steps facilitate an analysis process that allows the researcher to construct new theories instead of simply collecting data to test how well an established theory applies to the social phenomena they are studying. Theoretical sampling joined with constant comparative analysis raises the conceptual levels of data analysis and directs ongoing data generation [54].

Results will be discussed until consensus between analysts is achieved. The construction process of these thematic categories and codes is both inductive and deductive because the development of themes and subthemes rests on both literature and emerging categories of empirical analysis [53-55,67,71,73,74,77,78]. Ongoing analysis and recruitment will be undertaken until saturation of themes is reached, which Hennink et al [79] refer to as code saturation. Interpretations of the findings will be supported by direct quotes from both FGDs and individual IDIs.

FGDs usually yield both qualitative and observational data where analyses can be demanding. For the discussion, thematic analysis, as the most common forms of analysis in qualitative research, will be used to analyze the data collected. It puts emphasis on, pinpoints, examines, and records patterns (or “themes”) within the data [80]. Although the main source of data analysis is the recorded spoken language derived from the interview; nevertheless, reflection about the interview, the settings, and capturing the nonverbal communication expressed by the members of the groups would add a valuable dimension to the construction and analysis of data [81]. Prior to analysis, transcripts for each participant will be deidentified to ensure confidentiality and to limit analytical bias among researchers [82]. Qualitative data will be recorded, anonymously, integrally transcribed, and imported into the NVivo 12 software (QSR International), which will assist in the organization of the data during the analysis stage.

### Ethics Approval

The study obtained ethics approval and gatekeeper permission from the University’s Ethics Committee (Ref: BREC/00002153/2020) and the KwaZulu-Natal Provincial Department of Health (NHRD Ref: KZ_202010_030), as the study is to be conducted in health facilities in KwaZulu-Natal province. Written permission has been further sought from the concerned authorities and authorities from the different study sites to gain access to the facilities.

### Risks

This study is anticipated to involve minimal risks. This means that the probability of harm or discomfort anticipated as a result of participation in the study is not greater than those ordinarily encountered in the daily lives of the participants. Adequate care will be taken to ensure the competence of the fieldworker for the study through continued educational sessions.

### Potential Benefits

Participation in this research project will contribute to the improvement of collective medical, palliative, and public health knowledge that will enable the design of interventions to strengthen care coordination for patients with lung cancer while strengthening the cancer care program. The study will also provide strategic and scientific information on the implications of lung cancer during the access, diagnosis, referral, and treatment processes.

### Informed Consent

Informed consent is sought in writing from every potential participant prior to participating in the study. Both the English and isiZulu versions of the participant information sheet will be appended to the protocol. The informed consent forms comprise 2 sections: the participant information sheet and the informed consent. These are shared with each potential participant a day or 2 before data collection to allow them time to read and process the documents. Measures are taken to ensure that the respect, dignity, and freedom of each participant are honored in the study. To guarantee the anonymity of each participant, the names of respondents, their addresses, or other identifying information will not be included in the transcripts, but a unique identification number will be used. Further, participants will be assured of the confidentiality of all their information. Their rights to refuse to participate and to opt out of the study at any time will be emphasized.

## Results

This study receives support from the Multinational Lung Cancer Control Program, which is funded by Bristol-Myers Squibb Foundation. The support is granted for the lifespan of the study, from June 2020 to March 2023. The study obtained ethics approval and gatekeeper permission from the University’s Ethics and KwaZulu-Natal Provincial Department of Health, as the study is being conducted in health facilities in KwaZulu-Natal province. As of January 2023, we enrolled 50 participants, both health care providers and patients. We started with data analysis of interviews with patients and their caregivers, saturation was reached at 21 interviews. We have concluded data collection and analysis of interviews with health care providers, and finalizing manuscript write-up. Once all the data are analyzed, the results will be consolidated into a coherent report for dissemination. In addition, manuscripts for all phases will be prepared for publication. A scoping review article emanating from results from phase 2 was published and is currently working on the next step which is a systematic review, assessing the effectiveness of the identified coordination model.

## Discussion

### Anticipated Findings

Cancer is a complex condition that often requires multiple interventions provided by a variety of health professionals within the health care continuum [14-21]. The identification of current obstacles has the potential to guide the development of a model to improve the quality of coordinated cancer health care. Care coordination strategies are of great interest as they have the potential to improve the quality of health care, effectiveness, and optimal patient health outcomes. Care coordination requires effective communication and consistent transfer of health information between the different levels of care [27,32,33,37,52,83-88]. There is adequate literature supporting the need for and benefit of care coordination for people affected by cancer [14,19,27,33,34,38,39,47,89-94]. Care coordination is a set of activities needed to minimize the dangers of fragmentation [95], including the sharing of important clinical information by all health care providers involved with clear, shared expectations about their roles in the patient care process [49,51,52,95]. They also include efforts to keep patients and families informed and engaged throughout the care process. People with cancer are particularly at risk of receiving poorly organized and fragmented care due to the complex nature of the disease and its management [1,10,14,19,50,96-105]. Consequently, there is an increasing body of evidence on the potential of the patient navigation approach as a coordinated cancer care model for the effective improvement of access to health care services, especially for the poor, the uninsured, and other medically underserved populations [29,32,96,104-112]. The inclusion of patient navigation services in high-income countries is associated with improvements in access to timely diagnosis, treatment, and follow-up, among other quality indicators [11,85,112-115], something that is lacking in the LMICs [12]. In addition, evidence from global cancer agencies shows that improving access to clinical preventive services like cancer screening, linkage activities, and coordinated care among multiple providers not only minimizes delays and confusion about conflicting care plans but can also potentially reduce and prevent the disease [116].

There is a need to continuously review multilevel barriers (primary, secondary, and tertiary care) that threaten cancer care coordination across different settings and levels of clinical care to identify potential solutions for improving care. This study carries the promise of generating pertinent information for both policy makers and health care professionals. Decision makers will be provided with important information, concerning the proposed model designed with a costing exercise to determine the implementation viability. Once all the study phases are complete, the results will be consolidated into a coherent report for dissemination to key stakeholders and will also be presented in conferences and seminars. A technical report or policy brief is also anticipated from this study. A meeting of the relevant stakeholders will be organized, where the results will be presented and discussed. The thesis will be one of the outputs of this study, and this will be available in the University of KwaZulu-Natal library. The findings of the study will also be published in Department of Higher Education and Training accredited peer-reviewed journals.

The results of this study may be biased toward those patients who proactively seek or positively respond to diagnostic and treatment referrals by the health care professionals at the lower levels of care. Since the participating hospitals and the community-level study sites are located within the 2 major cities in KwaZulu-Natal, the profiles of patients may not be fully reflective of the geographical spread of the province. In addition, the global pandemic has presented a variety of challenges, including physical, emotional, financial, and much more, the effects of which are only beginning to be felt [117]. In our roles as researchers, these circumstances require us to be alert, innovative, and flexible in our approaches to collecting and disseminating data. The COVID-19 pandemic has disrupted the way that research is conducted [117] to the point that alternative ways of conducting research have become necessary. We hope to be able to come together virtually, collect and analyze data, rapidly generate knowledge, and disseminate it widely to improve the care of patients with cancer. Fortunately, the increased engagement of users on the internet and social media during the pandemic brings opportunities to engage in research through novel strategies and methods [118]. The results will be written up for publication in open-access, peer-reviewed journals for access by the scientific community, and abstracts will be presented at different conferences locally and internationally to encourage dissemination of the results. Shared information will be anonymized to ensure confidentiality.

### Conclusions

This study will provide comprehensive data to inform and empower patients, professionals, policy architects, and related decision makers to manage and improve cancer care coordination. This unique intervention will be designed to address the multifactorial problem of cancer health disparities. If successful, this study will help inform the design and implementation of coordination programs in LMICs to promote optimal cancer care for underserved patients.

## Abbreviations

ABC: activity-based costing
FGD: focus group discussion
GT: grounded theory
IDI: in-depth interview
LMIC: low- and middle-income country
